# Proniosomal gel-derived niosomes: an approach to sustain and improve the ocular delivery of brimonidine tartrate; formulation, *in-vitro* characterization, and *in-vivo* pharmacodynamic study

**DOI:** 10.1080/10717544.2019.1609622

**Published:** 2019-05-15

**Authors:** Alaa Emad Eldeeb, Salwa Salah, Mahmoud Ghorab

**Affiliations:** Department of Pharmaceutics and Industrial Pharmacy, Faculty of Pharmacy, Cairo University, Cairo, Egypt

**Keywords:** Glaucoma, brimonidine tartrate, sustained ocular delivery, proniosomal gel, tonometer

## Abstract

Brimonidine tartrate (BRT) is a hydrophilic α_2_ adrenergic agonist used for the treatment of glaucoma. Glaucoma is an ocular disease affecting the anterior segment of the eye requiring lifetime treatment. Owing to the obstacles facing ocular delivery systems and hydrophilicity of BRT, frequent administration of the eye drops is required. Niosomes have been widely used to improve the ocular bioavailability of the topically applied drugs and to enhance the ocular residence time. However, they have drawbacks as physical instability, aggregation, and loss of the entrapped drug. For this reason, BRT proniosomes were prepared to overcome niosomal instability issues. A D-optimal design was utilized to determine the optimum conditions for preparation of the proniosomal gels. Independent variables were amount of surfactant, surfactant:cholesterol ratio, and type of surfactant used. The dependent variables were entrapment efficiency (EE%), particle size, percentage of drug released after 2 h (Q_2h_), and percentage of drug released after 24 h (Q_24h_). The optimum formula was suggested with desirability 0.732 and the composition of 540 mg Span 60 and 10:1 surfactant:cholesterol ratio. The results obtained after reconstitution were; EE% of 79.23 ± 1.12% particle size of 810.95 ± 16.758 nm, polydispersity index (PDI) 0.6785 ± 0.213, zeta potential 59.1 ± 0.99 mV, Q_2h_40.98 ± 1.29%, Q_8h_ 63.35 ± 6.07%, and Q_24h_ = 91.11 ± 1.76%. Transmission electron microscope imaging of the formula showed the typical spherical shape of niosomes. *In-vivo* pharmacodynamic study assured the improved ocular bioavailability of BRT selected formula when compared with Alphagan^®^P with relative AUC_0–24_ of 5.024 and 7.90 folds increase in the mean residence time (MRT). Lack of ocular irritation of the formula was assured by Draize test.

## Introduction

Glaucoma is a serious disease affecting the anterior chamber of the eye (Wu et al., 2019). Normally aqueous humor is produced to nourish the anterior ocular tissues and drains through the trabecular meshwork and uveoscleral outflow. Imbalance occurs due to fluid overproduction or blockage of the drainage system leading to an accumulation of aqueous humor in the anterior chamber, this accumulation leads to an increase in the intraocular pressure (IOP). The elevation in IOP leads to an impairment in retinal blood flow, resulting in progressive degeneration of the optic nerve, causing gradual irreversible loss of vision (Quigley, 2005; Weinreb et al., 2014; Kapetanakis et al., 2016). Glaucoma symptoms include blurred vision, headache, hallows in vision, and progressive loss of vision. It is the most serious ocular disease requiring a lifetime treatment to prevent symptoms and progression of the disease (Mishra & Jain, 2014; Zeng et al., 2018). Pharmacological treatment of glaucoma based on either decreasing production of aqueous humor or increasing its drainage to restore the balance and prevent the buildup of aqueous humor.

Brimonidine tartrate (BRT) is a hydrophilic drug used for treatment of glaucoma, its pharmacological classification is α_2_ adrenergic agonist acts by dual mechanisms, decreasing aqueous humor production and increasing the uveoscleral outflow as it promotes prostaglandins synthesis, it has a neuroprotective action and decrease the ischemia-induced optic nerve damage (Saylor et al., 2009; Barse et al., 2018). Moreover, it has the advantage of being effective in normal tension glaucoma (Gandolfi et al., 2003), where IOP measurements are below 21 mm Hg but characteristic glaucomatous structural and functional optic nerve defects are present (Razeghinejad & Lee, 2019). It has no cardiovascular or pulmonary side effects caused by other glaucoma treatments (Timolol, betaxolol). BRT eye drops are taken in three times daily dose for entire life. The high dosing frequency is due to poor ocular bioavailability and leads to an impaired patient compliance.

Ocular drug delivery is highly challenging, only 1–3% of the instilled drug enters the eyes (Ghate & Edelhauser, 2006). Eye drops are the most used ocular dosage form (Song et al., 2018), however, they have major drawbacks that hinder effective drug delivery, they suffer from very short ocular residence time due to natural blinking, small capacity of the ocular surface, normal tear turnover, induced lacrimation, and nasolacrimal drainage (Khalil et al., 2017). Also, the drugs suffer from enzymatic degradation by the metabolic enzymes present in the tear film and corneal epithelial cells. In order to improve the ocular residence time, ocular delivery systems are developed as ocular inserts, gels, and *in-situ* gels, but they cause ocular discomfort and blurred vision (Prasannan et al., 2018).

Nano vesicular systems are being investigated for the last two decades to enhance the ocular bioavailability of the topically instilled drugs, they prolong the contact time between the drug and the ocular surface, as well as they provide a sustained manner of drug delivery, thus, enhancing the ocular bioavailability (Lalu et al., 2017). This was achieved by loading the drugs into liposomes (Abdelbary, 2011), niosomes (Abdelbary & El-gendy, 2008), cubosomes (Gan et al., 2010) spanlastics (Kakkar & Kaur, 2011), nanoemulsions (Ammar et al., 2009), and solid lipid nanoparticles (Yousry et al., 2016). Niosomes are nanovesicles formed by the use of nonionic surfactants. They have several advantages including (i) improved ocular residence time by its mucoadhesive nature, (ii) enhanced corneal permeation, and (iii) they are more advantageous than liposomes in that niosomal bilayer is formed from nonionic surfactants while liposomal bilayer is composed of phospholipids which are susceptible to oxidative degradation. Moreover, nonionic surfactants are cheaper, more available alternatives for natural phospholipids (Louis, 2018). Niosomes have certain limitations too, such as physical instability, aggregation, leakage of the entrapped drug, hydrolysis of encapsulated drugs, thus limiting the shelf life of the dispersion. To overcome these disadvantages, proniosomes are prepared and reconstituted into niosomes on hydration (Yadav et al., 2010).

Proniosomes are either proniosomal gels or dry granular proniosomes (Sudhamani et al., 2010). They minimize problems of niosomal dispersions (Radha et al., 2013). (i) Proniosomes solve the physical instability issues as aggregation, fusion and, leakage. (ii) They provide additional convenience in transportation, distribution, and storage (Yasam et al., 2014). (iii) Proniosomes can be used for entrapping both hydrophobic and hydrophilic drug with higher entrapment efficiency (EE%) (Blazek-Welsh & Rhodes, 2001). (iv) A comparative study conducted on stability studies of niosomes and proniosomes demonstrated that proniosomes can be effectively stored at room temperature and the drug leakage from the proniosome vesicles was reduced which is the main concern with niosome when stored at room temperature (Natesan et al., 2017). Proniosomal gels are chosen for our study over dry proniosomes as their method of preparation is simple, less time consuming, and no special equipment is required (Rajabalaya et al., 2016). Proniosomal gels are prepared using coacervation phase separation technique. It depends on the idea that the mixture of alcohol, surfactant, lipids, and aqueous phase can form concentrated proniosomal gel, which can be easily converted to stable niosomal dispersion by dilution with an excess amount of aqueous phase (Ishii et al., 1995). Nonionic surfactants are the vesicle forming agent. Cholesterol was added as it improves the rigidity of the vesicles by being intercalated between the bilayers of the nonionic surfactant, forming less leaky vesicles (Sambhakar et al., 2017). Lecithin is generally regarded as a membrane stabilizer, forming a tightly packed bilayer (Singh et al., 2015) hence, it improves the entrapment of the vesicles, also, it acts as a penetration enhancer (Yadav et al., 2010). Accordingly, the aim of our study is to explore the potential use of proniosomal gel derived niosomes in ocular delivery of BRT to improve the ocular bioavailability of the drug, prolong the ocular residence time, and to attain sustained drug release.

## Materials and methods

### Materials

BRT was kindly supplied by Orchidia pharmaceutical company, Obour, Egypt. L-α-phosphatidylcholine from soya bean type IV-S (enzymatic), Cholesterol, Brij 52, Dialysis membrane 12,000–14,000 molecular weight cut off were purchased from Sigma-Aldrich Co. (Darmstadt, Germany). Span 60 (Sorbitan monostearate) was purchased from SD fine chemicals, Mumbai, India . Ethyl alcohol (95%), Tween 80, methanol, sodium chloride, sodium bicarbonate, disodium hydrogen phosphate, potassium dihydrogen orthophosphate, and anhydrous calcium chloride were purchased from El Nasr pharmaceutical company (Cairo, Egypt). The market eye drops Alphagan^®^P (batch no. 95184) manufactured by Allergan (Allergan Inc., Irvine, CA) was purchased to be used in comparative studies.

### Methods

#### Determination of the optimum conditions for preparation of BRT-loaded proniosomal gels

##### Preliminary screening

In order to investigate the better choice of a surfactant to be used in the preparation of BRT loaded proniosomal gels, two surfactants were utilized. Tween 80 as a representative of a surfactant having a high hydrophilic–lipophilic balance (HLB) value (HLB = 15) and Span 60 as a representative of surfactant with a low HLB value (HLB = 4.7). Also, lecithin amount was varied with only two levels; 180 and 360 mg. The effect of varying those two independent variables on EE%, particle size, percentage of drug released after 2 h (Q_2h_), and percentage of drug released after 8 h (Q_8__H_) of their derived niosomes were investigated. Four formulae were prepared and characterized. The composition of the formulae is represented in Table 1(a).

**Table 1.** The composition of the investigated brimonidine tartrate loaded proniosomal gels.

(a) Preliminary screening and the results obtained after reconstitution.								
Formula^a^	Lecithin amount (mg)	Type of surfactant	Entrapment efficiency (%)^b^	Particle size (nm)^b^	PDI^b^	Zeta potential (mV)^b^	Q2h(%)^b^	Q8h (%)^b^
PN1	180	Tween 80	55.6±1.9	45.8±1.9	0.561±0.04	−49.3±0.4	61.9±3.1	96.19±0.1
PN2	360	Tween 80	44.6±0.1	98.9±1.4	0.251±0.001	−49.8±0.3	62.6±0.5	91.15±2
PN3	180	Span 60	73.5±3.7	695.4±35.6	0.644±0.22	−66±2.6	68.59±1.3	85.99±1.9
PN4	360	Span 60	83.4±2.3	1277.5±154.9	0.262±0.22	−73.45±1	61.1±2.5	72.1±3.4

**Table:** 

(b) Composition of the formulae according to the D-optimal design and the results obtained after reconstitution.										
Formula^c^	X1: Amount of surfactant (mg)	X2: Ratio of surfactant: cholesterol	X3: Type of surfactant	Y1: Entrapment efficiency (%)^b^	Y2: Particle Size (nm)^b^	PDI^b^	Zeta potential (mV)^b^	Y3: Q2h (%)^b^	Q8h (%)^b^	Y4: Q24h (%)^b^
F1	180.00	2.00:1.00	Span 60	64.88 ± 12.93	829.90 ± 91.92	0.490 ± 0.214	−63.75 ± 2.05	58.37 ± 4.57	88.18 ± 2.53	100.41 ± 1.91
F2	180.00	2.00:1.00	Span 60	67.87 ± 13.98	742.80 ± 6.93	0.374 ± 0.105	−54.30 ± 0.28	61.94 ± 3.22	87.96 ± 11.48	97.85 ± 4.19
F3	180.00	10.00:1.00	Span 60	68.35 ± 5.22	587.40 ± 0.42	0.536 ± 0.060	−67.20 ± 0.42	56.82 ± 1.93	84.17 ± 2.96	97.47 ± 5.09
F4	272.82	6.52:1.00	Span 60	73.88 ± 5.68	739.75 ± 37.27	0.481 ± 0.153	−68.85 ± 2.61	59.10 ± 1.57	82.54 ± 3.22	93.08 ± 6.42
F5	360.00	2.25:1.00	Span 60	86.48 ± 8.51	1206.00 ± 41.01	0.268 ± 0.148	−76.85 ± 2.48	33.45 ± 7.82	60.44 ± 13.48	79.76 ± 8.78
F6	360.00	10.00:1.00	Span 60	83.20 ± 6.54	668.85 ± 106.99	0.700 ± 0.187	−63.70 ± 1.84	43.26 ± 1.61	67.80 ± 6.44	78.12 ± 14.64
F7	455.59	6.00:1.00	Span 60	93.71 ± 1.83	796.85 ± 110.66	0.528 ± 0.158	−69.75 ± 0.64	38.53 ± 5.89	69.23 ± 7.75	75.08 ± 10.99
F8	540.00	2.00:1.00	Span 60	85.48 ± 0.68	1313.00 ± 367.70	0.572 ± 0.235	−67.65 ± 0.07	35.84 ± 3.14	61.48 ± 8.85	75.08 ± 12.99
F9	540.00	2.00:1.00	Span 60	85.60 ± 0.88	2018.00 ± 422.85	0.501 ± 0.241	−72.25 ± 1.77	35.54 ± 0.72	54.85 ± 5.19	75.24 ± 5.57
F10	540.00	10.00:1.00	Span 60	84.83 ± 0.14	1177.50 ± 251.02	0.564 ± 0.117	−64.85 ± 0.64	40.30 ± 2.19	65.78 ± 11.42	85.60 ± 6.90
F11	540.00	10.00:1.00	Span 60	82.63 ± 0.30	1000.95 ± 282.91	0.937 ± 0.087	−62.10 ± 0.85	38.15 ± 0.53	75.44 ± 0.75	90.57 ± 1.20
F12	180.00	5.93:1.00	Brij 52	40.40 ± 4.29	345.55 ± 3.75	0.242 ± 0.005	−62.40 ± 2.83	69.34 ± 2.93	85.58 ± 3.39	99.92 ± 1.56
F13	180.00	5.93:1.00	Brij 52	46.03 ± 2.99	310.80 ± 1.13	0.441 ± 0.094	−63.15 ± 3.89	75.24 ± 0.49	90.35 ± 0.18	95.18 ± 1.51
F14	356.70	10.00:1.00	Brij 52	46.46 ± 0.00	398.85 ± 5.73	0.469 ± 0.008	−61.65 ± 1.77	51.28 ± 11.53	69.12 ± 0.60	91.56 ± 4.14
F15	356.70	10.00:1.00	Brij 52	46.68 ± 6.13	430.90 ± 4.38	0.438 ± 0.035	−62.35 ± 0.35	57.73 ± 0.54	83.71 ± 6.57	91.99 ± 8.06
F16	360.00	6.00:1.00	Brij 52	55.92 ± 2.01	495.45 ± 1.49	0.423 ± 0.044	−60.45 ± 3.18	54.35 ± 3.88	73.35 ± 0.60	79.71 ± 1.92
F17	362.74	2.00:1.00	Brij 52	55.58 ± 1.64	828.65 ± 9.83	0.366 ± 0.252	−60.60 ± 0.42	63.52 ± 13.51	79.27 ± 14.98	85.36 ± 9.99
F18	540.00	2.00:1.00	Brij 52	44.41 ± 2.91	559.95 ± 7.57	0.599 ± 0.220	−55.80 ± 2.40	61.50 ± 1.40	72.68 ± 0.60	75.20 ± 0.86
F19	540.00	6.09:1.00	Brij 52	43.90 ± 12.93	498.65 ± 9.97	0.403 ± 0.033	−54.70 ± 1.84	44.03 ± 0.53	65.48 ± 1.24	68.95 ± 0.80

^a^Prepared proniosomes contained 0.15% of brimonidine tartrate, 540 mg of a surfactant, and the aqueous phase is composed of phosphate buffered saline pH 7.4.

^b^Data presented as mean ± SD (*n* = 3).

^c^Prepared proniosomes contained 0.15% of brimonidine tartrate, 360 mg lecithin, and the aqueous phase is composed of phosphate buffered saline pH 7.4.

PDI: polydispersity index; Q2h, Q8h, Q24h: percentage of drug released after 2, 8, and 24 h, respectively.

##### Implementation of a D-optimal design to investigate the best criteria to prepare BRT-loaded proniosomal gels

Based on the analysis of the results obtained from the preliminary screening, the implementation of a D-optimal design was done using Design-Expert^®^ software (Stat-Ease, Inc., Minneapolis, MN). Studying the effect of three independent variables namely, amount of surfactant, surfactant:cholesterol ratio, and type of surfactant used (two hydrophobic surfactants with low HLB values were used, Span 60 (HLB = 4.7) and Brij 52 (HLB = 5.3)). Levels of the independent variables are represented in Table 2(a). The composition of the 19 formulae suggested by Design-Expert^®^ is shown in Table 1(b). Those independent variables were chosen to investigate their effect on the dependent variables; EE%, particle size, Q2h, and Q24h.

**Table 2.** (a) D-optimal design factors, their levels, and target constraints.

Independent variables (Factors)	Lowest level	Highest level
Amount of SAA (mg) (A)	180	540
Ratio of SAA:cholesterol (B)	2:1	10:1
Type of the surfactant used (C)	Span 60	Brij 52
Dependent variables (responses)	Constraints	
Entrapment efficiency % (Y1)	Maximize	
Particle size (nm) (Y2)	Minimize	
Q2h (Y3)	Minimize	
Q24h (Y4)	Maximize	

**Table:** 

(b) Statistical analysis of the D-optimal design.							
Response	Model	*R*^2^	Adjusted *R*^2^	Predicted R^2^	Adequate precision	Model equation in terms of coded factors	Significant model terms
Entrapment efficiency	Reduced quadratic	0.9829	0.9720	0.9512	23.287	Entrapment efficiency = +70.21 + 4.89 *A - 2.40 * B-16.88 * C-4.83 * A * C-1.70 * B * C-9.62* A^2 - 2.19 * B^2	A, B, C, AC, A^2^
Particle size	Linear	0.7677	0.7213	0.6242	11.183	Particle size = +684.16 + 154.42* A-94.92 * B -199.90 * C	A, B, C
Q2h	Linear	0.7821	0.7385	0.6578	12.133	Q2h = +53.07 − 10.56 * A -1.44 * B +6.53* C	A,C
Q24h	Linear	0.8268	0.7602	0.6636	10.997	Q24h = +80.92 -10.83 * A +3.62 * A * B -2.25* A * C +3.70 * A^2 + 5.04 * B^2	A

SAA: surfactant; A: Amount of surfactant (mg); B, SAA: cholesterol ratio; C: Type of surfactant.

#### Preparation of BRT loaded proniosomal gels according to coacervation phase separation method

Proniosomes were prepared using coacervation phase separation method reported by Perrett et al. (1991) and Vora et al. (1998). For each formulation, accurately weighed amounts of α-lecithin from soya bean, cholesterol, surfactant, and BRT were mixed with 2.5 ml of ethyl alcohol (95%) in a small stoppered glass vial. The glass vial was added to a water bath at a temperature of 70–80°C for 30 min with frequent shaking until complete dissolution of lipids. Then preheated 0.9 ml of phosphate buffer saline (PBS) was added to the molten lipids mixture and left on the water bath for 10 min till the mixture becomes a clear solution. The mixture left to cool down to room temperature for 24 h and the white creamy proniosomal gel was formed (Fouda et al., 2018). When needed, the proniosomal gel is reconstituted with PBS and sonicated to form BRT loaded niosomes immediately.

#### Compatibility of brimonidine tartrate with the used additives using Fourier-Transform infrared (FTIR) spectroscopy

FTIR spectra for BRT, 1:1 physical mixture with each of the ingredients, and the physical mixture of the optimized formulation components were recorded at ambient temperature using FTIR-8400 (Shimadzu, Kyoto, Japan). Approximately, 2–3 mg of each sample was compressed into a disc by mixing with dry potassium bromide then scanned at the scanning range of 4000–400 cm^−1^.

#### *In-vitro* characterization of BRT loaded proniosomal gel-derived niosomes

Each proniosomal gel formulation is reconstituted with PBS to form 10 ml of BRT loaded niosomes. The formed niosomes are then used for further characterization.

##### Determination of BRT entrapment efficiency percent (EE%)

EE% was determined indirectly by calculating the difference between BRT left in the supernatant after centrifugation of the formula and the total amount of BRT added. An aliquot of 1 ml of the formula was centrifuged at 18,000 rpm for 1 h at 4 °C using cooling ultracentrifuge (Sigma 3-30 KS, Sigma Laborzentrifugen GmbH, Germany). The supernatant obtained after centrifugation was appropriately diluted with methanol and the amount of unentrapped BRT was measured spectrophotometrically (model UV-1601 PC, Shimadzu, Kyoto, Japan) using methanol as a blank at λ_max_ = 320 nm. Drug EE% was determined according to the following equation (Habib et al., 2018):

$$\text{EE\% }=\;\frac{\left(\text{Total amount of BRT}-\text{Amount of unentraped BRT}\right)}{\text{Total amount of BRT}}\times 100$$

##### Determination of particle size, polydispersity index (PDI) and zeta potential of the prepared formulae

The dispersions were properly diluted with distilled water and measured by Malvern Zetasizer (Malvern Instruments Ltd., Worcestershire, UK) to determine particle size, polydispersity index **(**PDI), and zeta potential of the prepared formulae.

##### *In-vitro* release and kinetic analysis of the release data

The *in-vitro* release of the formulae and Alphagan^®^P was done in a horizontal shaking water bath (Gesellschatt laboratories, Berlin, Germany) adjusted at 50 rpm agitation speed and a temperature of 35 ± 0.5 °C. A bottle containing 50 ml of the release medium which was simulated lacrimal fluid pH 7.4 (SLF) (NaCl 0.67 g, NaHCO_3_ 0.20 g, CaCl_2_.2H_2_O 0.008 g, and distilled water to 100 ml (Marques et al. n.d.)) was added to the shaker and a presoaked dialysis bag (12,000-14,000 molecular weight cut off) containing 2 ml of the tested formula was immersed in the release medium inside the glass bottle. At predetermined time intervals, aliquots of 1 ml were withdrawn from the release medium and replaced by one ml of fresh release medium to maintain a constant volume (Fouda et al., 2018).

The percentage of BRT released at each time interval was spectrophotometrically measured at the predetermined *λ*_max_ *(λ* = 320 nm) using SLF as a blank and the % drug released at each time was calculated using the following equation (Teodorescu et al., 2017; Habib et al., 2018):

$$Qn\;=\;\frac{Cn\times Vr+\sum_{i=1}^{n-1}\mathrm{Ci}\times Vs}{\text{Initial drug amount}}$$

where
  Q*n*: Current cumulative percent of drug released
C*n*: The receptor medium current concentration at nth sample
*V*r: Receptor medium volume
*V*s: Volume of each sample removed for analysis
$\sum_{i=1}^{n-1}\mathrm{Ci}:$ Summation of the previously measured concentrations.

Results are the mean values of the release experiments (*n* = 3). The release profiles were drawn by plotting the percentage of drug released (Q*_n_*) at each time point *v*s time.

Data obtained from the release of the drug from different formulae were kinetically analyzed using Excel 2010^®^ (Microsoft, software, Redmond, WA) to determine the mechanism and the order of drug release. Generally, zero order kinetics, first order kinetics, second order, third order, and Higuchi’s diffusion models were used for the analysis of the release data.

### Statistical analysis

Analysis of the results obtained from the formulae suggested by the D-optimal design was done using Design-Expert^®^ software to determine the influence of the chosen variables on EE%, particle size, Q2h, and Q24h.

### Optimization of the conditions for preparation of BRT loaded proniosomal gel using D-optimal design for the selection of the best formula

After analysis of the results obtained from the design, Design-Expert^®^ software was used to choose the optimum conditions for preparation of BRT loaded proniosomal gel based on desirability function. The software allowed consideration of all responses at the same time. Maximizing EE% and Q24h as well as minimizing the particle size and Q2h were the common targets for preparation. Optimized formula with the highest desirability was prepared and tested for the forementioned *in-vitro* characterizations. Also, it was utilized for further investigations.

### Further characterizations for the selected optimized formula

#### Imaging by transmission electron microscope (TEM)

To visualize the morphological features of the prepared proniosomal gel-derived niosomes, one drop of the optimum formulation after reconstitution into niosomes was appropriately diluted and adsorbed on a carbon coated copper grid. A filter paper was used to remove the excess of the dispersion, then left to dry for 10 min at room temperature and examined by TEM (Joel JEM 1400, Tokyo, Japan).

#### Effect of gamma sterilization

The optimized formula was sterilized terminally by gamma irradiation arising from ^60^Co irradiator with 10 kGy irradiation dose (Volland et al., 1994; Morsi et al., 2019) (National Center for Radiation Research and Technology, Nasr City, Egypt). EE%, particle size, zeta potential, PDI, Q2h, and Q24h were compared before and after sterilization using one way ANOVA test (at *p*<.05). Release profiles of the optimized formula before and after sterilization were compared by calculating similarity factor (*f*_2_) using the following equation (Flanner, 1996; Fahmy et al., 2018):

$$f2=50\log\left\{{\left[\left.1+\left(\frac{1}{n}\right)\right.\sum_{t=1}^{n}{\left(R_{t}-T_{t}\right)}^{2}\right]}^{-0.5}\times 100\right\}$$

*R_t_*: Percentage of drug released from the optimized formula before sterilization at time *t.*
*T_t_*: Percentage of drug released of the optimized formula after sterilization at time *t*.

#### Differential scanning calorimetry (DSC)

Optimized formulation was frozen and lyophilized for 24 h at −45 °C under a pressure of 7 × 10^−2^ mbar using a lyophilizer (Novalyphe-NL 500, Savant Instruments, Holbrook, NY) (Fahmy et al., 2019). DSC analysis was performed for the lyophilized optimized formula and pure BRT. A Differential scanning calorimeter (Shimadzu DSC 50; Kyoto, Japan) was used for recording their corresponding DSC thermograms. Each sample (3–4 mg) was added to a flat-bottomed aluminum pan and heated at a constant rate 10°C/min, to a temperature of 400°C, under an inert nitrogen flow of 30 ml/min.

### *In-vivo* evaluation of the optimized BRT formula

#### Ocular irritation evaluation

Draize test is the most reliable test for determination of ocular irritation of prepared formulations (Draize et al., 1944). Seven New Zealand male albino rabbits were subjected to the administration of the optimized formula and the market eye drops in order to evaluate the irritancy of both (Shivakumar et al., 2007). Draize test depends on scoring system ranging from 0 (no irritation) to +3 (highest irritation and redness) for the cornea, iris, and conjunctivae. The tested formulation was applied in the conjunctival sac of the right eye and the left eye was kept as control by instillation of saline. The cornea, iris, and conjunctivae were examined for any signs of irritation or congestion caused by the formulation. Testing the ocular irritation score was done at intervals of 1, 2, 5, 8, and 24 h after administration (Tayel et al., 2013; Shokry et al., 2018).

#### *In-vivo* pharmacodynamic study for the evaluation of IOP lowering effect of the optimized formula

The protocol of the study (PT 1924) was presented to the Research Ethics Committee in the Faculty of Pharmacy, Cairo University (REC-FOPCU), Egypt. Study design was a single dose cross over design with a one week washout period.

### Procedures

The study design involved seven male New Zealand albino rabbits with healthy ocular features weighing from 2 to 2.5 Kg hosted in the animal house in the Faculty of Pharmacy Cairo University, Giza, Egypt. Rabbits were kept under standard conditions of animal housing, at an air-conditioned room with a temperature of 22 ± 0.5°C, an alternating light, and dark cycles, and were fed on standard diet and water. The tested eye drops were Alphagan^®^P (0.15%) and the optimized formula having the same concentration as the market product. The tested eye drops were applied in the lower cul de sac of the right eye and saline solution was applied in the left eye to be kept as a control, the eye was blinked three times after each instillation. IOP of the treated and the control eye was measured using SchiÖtz Tonometer (Rudolf Riester GmbH and Co. KG, Germany) at sequential intervals of 0.25, 0.5, 1, 1.5, 2, 3, 5, 8, and 24 h after instillation. The percentage decrease in IOP at each time interval was calculated using the following formula (Ammar et al., 2009):

$$\text{\% decrease in IOP }=\frac{\text{IOP control eye}-\text{IOP treated eye}}{\text{IOP control eye}}$$

Data obtained from the experiment were entered in Kinetica VR software 2000 (Innaphase Corporation, Philadelphia, PA). Plots of % decrease in IOP against time for the seven rabbits were obtained and the following parameters were determined for each plot: maximum percentage decrease in IOP (% Dec. IOP_max_), Time for maximum percentage decrease in IOP (T_max_), mean residence time (MRT), and area under percentage decrease in IOP against time curve from zero to 24 h (AUC_0–24 h)_. The significance of the difference in these parameters between the selected formula and the market product was tested using SPSS software (SPSS Inc., Chicago, IL) (at *p* < .05).

## Results and discussion

### Determination of the optimum conditions for preparation of BRT-loaded proniosomal gels

#### Preliminary screening

Achieving sustained drug release from vesicles is accomplished by high entrapment of the drug. Results obtained from the preliminary screening are represented in Table 1(a). Analysis of the results showed that Span 60 based proniosomes give rise to more entrapped BRT and more sustained drug release than Tween 80 based ones. This is due to the increased hydrophobicity of the bilayers formed by Span 60 due to its longer alkyl chains and the lower HLB value of Span 60 (HLB = 4.7) leading to effective entrapment of the drug (Aburahma & Abdelbary, 2012). Tween 80 is a hydrophilic surfactant with a large polar head and a high HLB value (HLB = 15) forming a leaky bilayer. Also, Tween 80 contains a double bond in the alkyl chain. The presence of the double bond made the chains bend leading to the formation of a leaky membrane as the adjacent molecules cannot form a tight membrane. These results are in agreement with Abdelbary et al. (2017) and Hao et al. (2002). Span 60 based vesicles are larger than Tween 80 niosomes, this is in agreement with results obtained by Sambhakar et al. (2017). The higher amount of lecithin led to higher EE% and more sustained release of the drug as lecithin increases the Tc (phase transition temperature), decreasing the fluidity of the bilayer (Abdelbary et al., 2017).

#### Implementation of a D-optimal design to investigate the best criteria to prepare BRT-loaded proniosomal gels

Based on the analysis of the results obtained from the preliminary screening, we decided to implement a D-optimal design to study the effect of formulation variables on the *in-vitro* characterizations.

### Preparation of BRT loaded proniosomal gels according to coacervation phase separation method

Coacervation phase separation method is a simple technique for the preparation of proniosomes (Yasam et al., 2014). As mentioned in the introduction section, it is based on the ability of the used ingredients to form concentrated proniosomal gel, which can be reconstituted to stable niosomal dispersion (Ishii et al., 1995). Nonionic surfactants are the most commonly used surfactants in preparation of vesicles due to their compatibility, stability, and non-toxicity. Cholesterol and lecithin are generally regarded as a membrane stabilizers and penetration enhancers (Yadav et al., 2010). Nonionic surfactants, cholesterol, and lecithin are listed in GRAS and FDA inactive ingredients databases (Rowe et al., 2009). It is reported that alcohol type affects the size of the vesicles. Ethanol was chosen as it forms larger vesicles with higher entrapment when compared with other alcohols. The larger size with ethanol could be due to its greatest solubility in water which leads to slower phase separation (Singh et al., 2015).

### Compatibility of BRT with the used additives using Fourier-transform infrared (FTIR) spectroscopy

FTIR spectroscopy was done to assess the possible interactions between the drug and the other ingredients used in the preparation of proniosomal gels. FTIR spectra of BRT is shown in Figure 2(I–A) showing characteristic ─NH bending vibrations at 1593 cm^−1^, C═N, and C═C stretching vibrations at 1651 cm^−1^, and the C═O group stretching vibrations at 1732 cm^−1^ (Aburahma & Mahmoud, 2011). FTIR spectra of the physical mixtures of BRT with each of the ingredients alone and the physical mixture of the optimized formulation components are represented in Figure 2(I-B–F). Showing preservation of the characteristic bands of BRT after mixing, indicating the lack of interference with the used ingredients.

### *In-vitro* characterization of the BRT loaded proniosomal gel-derived niosomes

All results are listed in Table 1(b).

#### Entrapment efficiency percent (EE%)

**Figure 1.**
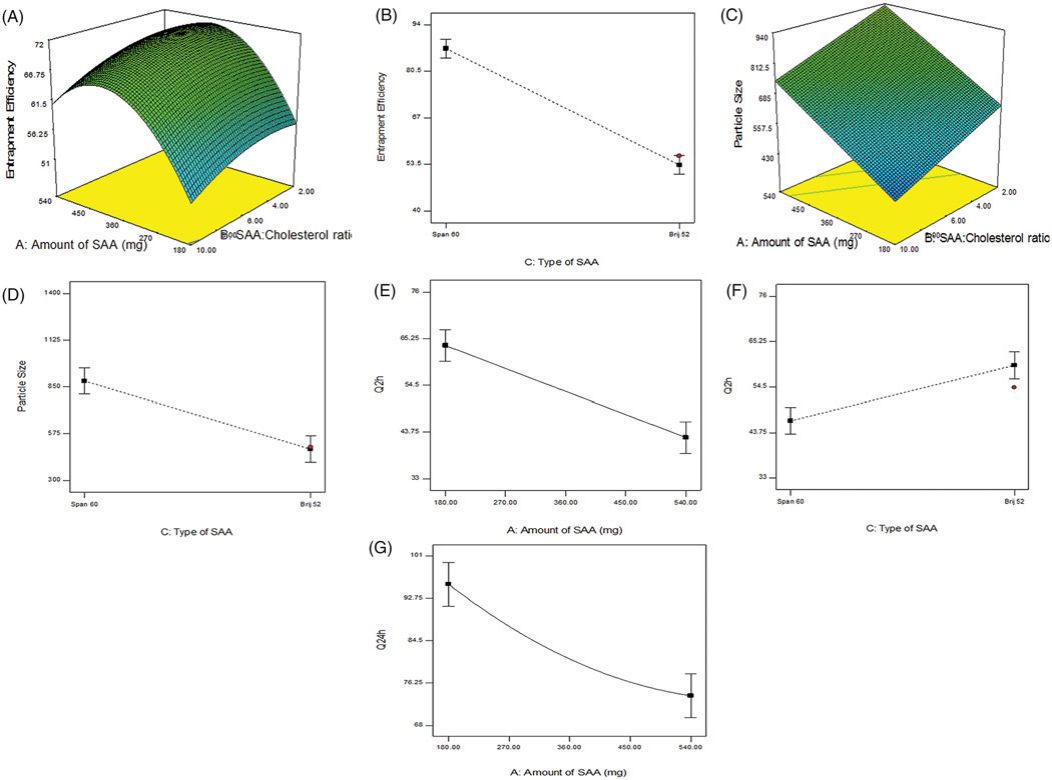
Response 3-D plots for the effect of amount of surfactant (SAA) (X1) and surfactant:cholesterol ratio (X2) on entrapment efficiency % (A), type of surfactant on entrapment efficiency % (B), amount of surfactant (X1) and surfactant:cholesterol ratio (X2) on particle size (C), type of surfactant on particle size (D), amount of surfactant on Q2h (E), type of surfactant on Q2h (F), type of surfactant on Q24h (G).

As shown in Table 1(b), entrapment efficiencies of Span 60 prepared formulae (F1-F11) ranged from 64.88 ± 12.93% to 93.71 ± 1.83% while Brij 52 based formulae (F12-F19) ranged from 40.40 ± 4.29% to 55.92 ± 2.01%. Statistical analysis of formulation variables on EE% (at *p*<.05) showed that A (surfactant concentration), B (surfactant:cholesterol ratio), and C (type of surfactant) have a significant effect on EE%. As shown in Figure 1(A), amount of surfactant has a positive significant effect on EE%, this could be justified by the more number of vesicles formed by the increased amount of surfactant (Thomas & Viswanad, 2012). Also, increasing the amount of surfactant leads to an increase in the hydrophobic domain, hence increases the entrapment of the drug (Ramkanth, 2018). However, further increase in surfactant amount showed a decrease in EE%, this could be due to the formation of mixed micelles together with niosomes, which may lead to lower EE% (Ramkanth, 2018). Figure 1(A) showed that increasing surfactant:cholesterol ratio leads to a decrease in EE%. This could be attributed to that at low surfactant:cholesterol ratio, cholesterol amount is high. Cholesterol increases the rigidity of the bilayer as it acts as vesicular cement, it promotes the hydrophobicity of the bilayer, so enhances the incorporation of BRT inside the vesicles. The increased rigidity hinders the leakage and permeability of the membrane (Abdelbary et al., 2017).

Figure 1(B) showed that Span 60 based proniosomal gels give rise to niosomes with more EE% than Brij 52 based ones. This could be attributed to the transition temperature of both surfactants, Span 60 being solid at room temperature has a higher phase transition temperature (Tc = 53 °C) than Brij 52 (Tc = 32.5 °C). The high transition temperature decreases the fluidity and leakage of the bilayer. Moreover, Span 60 has a lower HLB value (HLB = 4.7) with a longer C17 alkyl chain compared to Brij 52 (HLB = 5.3) and C16, this sequentially leads to better entrapment of hydrophilic drugs due to the increased hydrophobicity (Yoshioka et al., 1994; Ammar et al., 2017).

#### Particle size, PDI, and zeta potential

The particle size of the prepared proniosomes after reconstitution ranged from 310.8 ± 1.13 to 2018 ± 422.85 nm. Span 60 based formulae (F1-F11) ranged from 587.4 ± 0.424 to 2018 ± 422.85 nm, while Brij 52 based formulae (F12-F19) ranged from 310.8 ± 1.131 to 828.65 ± 9.83 nm. It is clear that all formulae having a particle size less than 5 µm which is suitable for ocular administration (Janoria et al., 2007; Fouda et al., 2018). Statistical analysis of formulation variables on EE% (at *p*<.05) showed that A (surfactant concentration), B (surfactant:cholesterol ratio) and C (type of surfactant) have a significant effect on particle size. As shown in Figure 1(C), amount of surfactant has a positive linear effect on particle size, this could be attributed to the increment in the entrapment of BRT, so resulting in larger vesicles (Manconi et al., 2002). Surfactant:cholesterol ratio has a negative linear effect on particle size. The decrease in the surfactant:cholesterol ratio from 10:1 to 2:1 resulted in an increase in the size of the vesicles. This may be attributed to the disturbance in the niosomal membrane imparted by the high amounts of cholesterol. Cholesterol is amphiphilic molecule being intercalated within the bilayer by orienting itself with the polar head toward the aqueous surface and the aliphatic chain line up parallel to the surfactant hydrocarbon chains, consequently, leading to the formation of larger vesicles (Essa, 2010). Span 60 based formulae have larger vesicular size than Brij 52, that could be justified by higher EE% of Span 60 based niosomes and the interaction between cholesterol and Span 60 leading to larger vesicles (Sambhakar et al., 2017). PDI of the formulae are represented in Table 1(b) and ranged from 0.374 ± 0.105 to 0.937 ± 0.087 indicating variable homogeneity of particles.

Zeta potential (ZP) is a critical parameter indicating the stability of colloidal dispersions (Dai et al., 2010). High ZP values indicate increased electrical repulsion forces between the particles, preventing their aggregation and coalescence. ZP of the formulae ranged from −54.30 ± 0.28 to −72.25 ± 1.77 mV. ZP values for each formula are represented in Table 1(b). These high values ensure good stability of the dispersions (White et al., 2007; Lal et al., 2011). Negative values of ZP are due to the ionization of the free hydroxyl groups present in surfactant and cholesterol (Zubairu et al., 2015). Also, lecithin is composed of phospholipids which on ionization at neutral pH contribute to their negative charge (Wang & Wang, 2008).

#### *In-vitro* release and kinetic analysis of the release data

As shown in Figure 3(A–C) prepared formulae showed variable release profiles over 24 h, while Alphagan^®^P showed 100% drug release within the first 2 h. *In-vitro* drug release gives an indication of how the drug will behave *in-vivo* (Hundekar et al., 2014). Niosomes are well known for being efficient vesicles for the sustainment of drug release with a unique biphasic pattern (Mokhtar et al., 2008). Initial burst effect in the first 2 h, due to the diffusion of the surface attached drug, then followed by a sustainment in the drug release, due to the diffusion of the entrapped drug from inside the vesicles to the surrounding aqueous medium. Factors that increase vesicular membrane rigidity (low HLB, high transition temperature, and long alkyl chain) lead to an increase in the entrapment of the drug and consequently lead to a more sustained profile. Also, the more hydrophobic the way, the harder will be the partitioning of the drug to the surrounding medium. These justifications explain the superiority of Span 60 based niosomes in sustainment of drug release when compared to Brij 52.

As shown in Figure 1(E,G) statistical analysis (at *p* < 0.05) showed that A amount of surfactant) has a negative significant effect on Q2h and Q24h. As mentioned in the discussion section of EE% results, the increased amount of surfactant leads to an increment in drug EE%. Moreover, the increased amount of surfactant leads to an increase in the viscosity of the dispersion, these reasons elucidate the slower release exhibited by the higher amounts of surfactant. While C (type of surfactant) has a significant effect on Q2h. Span 60 based proniosomes showed a lower Q2h when compared with Brij 52. This could be justified by the higher entrapment of Span 60 based formulations. The lower Tc of Brij 52 leading to formation of a leaky bilayer, facilitating the drug release. Kinetic analysis of the release data proved that the drug release mechanism from the prepared formulations follows Higuchi’s diffusion mechanism.

### Statistical analysis

Statistical analysis of the results and the best model fitting the data is represented in Table 2(b). Discussion of the significant factors is mentioned in the *in-vitro* characterization of the BRT loaded proniosomal gel-derived niosomes section.

### Optimization of the conditions for preparation of BRT loaded proniosomal gel using D-optimal design for the selection of the best formula

Based on target constraints, the optimum formula with desirability 0.732 was selected. Suggested composition of the formula is 540 mg Span 60 and 10:1 surfactant: cholesterol ratio. The formula was prepared and the results obtained were; EE% = 79.23 ± 1.12%, particle size of 810.95 ± 16.758 nm, PDI 0.6785 ± 0.213, zeta potential 59.1 ± 0.990 mV, Q2h 40.98 ± 1.29%, Q8h 63.35 ± 6.07%, and Q24h = 91.11 ± 1.76%. The *in-vitro* release profile is shown in Figure 3(D).

### Further characterizations for the selected optimized formula

#### Morphology of the optimized formula investigated by TEM

**Figure 2.**
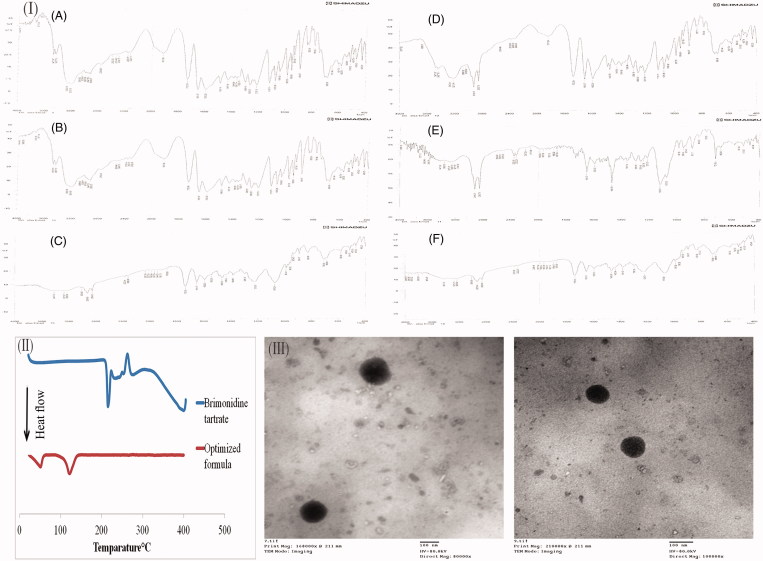
(I) The IR charts of brimonidine tartrate (BRT) (A), BRT:Cholesterol (B), BRT:Lecithin (C), BRT:Span 60 (D), BRT:Brij 52 (E), BRT:Cholesterol:Lecithin:Span 60 (F), (II) DSC thermograms of BRT and the lyophilized optimized formulation, (III) Transmission Electron Microscope images of the optimized formula.

The TEM micrographs of the selected formulation after reconstitution into niosomes are shown in Figure 2(III). The micrographs demonstrated that the niosomal vesicles are not aggregated and present in a typical spherical shape of niosomes.

#### Effect of gamma sterilization

**Figure 3.**
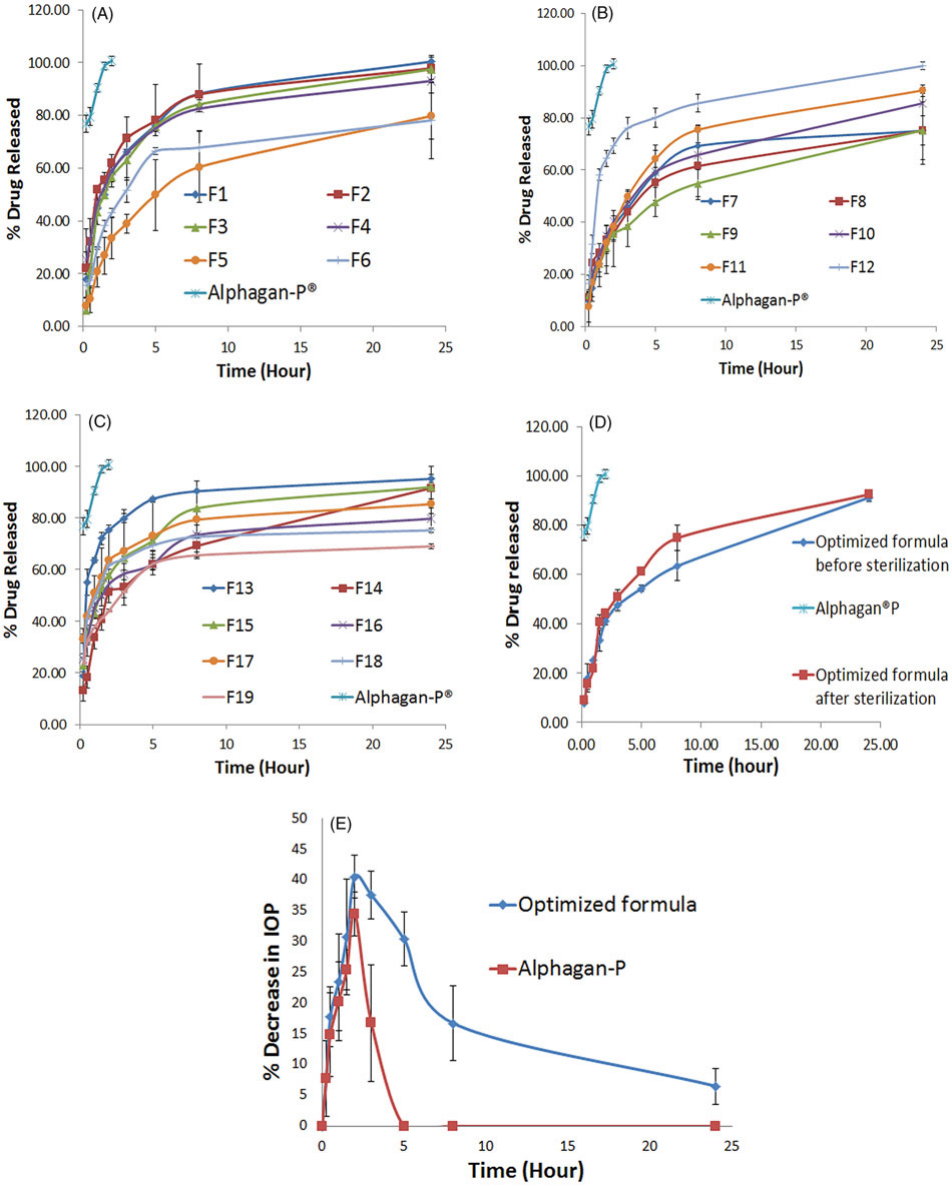
*In-vitro* release profiles of BRT loaded proniosomal gel-derived niosomal formulae in comparison with Alphagan^®^P (A–C), *In-vitro* release profile of the optimized formula before and after sterilization in comparison with Alphagan^®^P (D), *in-vivo* plot of the percentage decrease in IOP as a function of time response curve after ocular administration of optimized formula and Alphagan^®^P in albino rabbits (E).

Sterilization of ocular preparations is a must to prevent co-infection of the eye with microorganisms that might be present in the formulation. Sterilization of the selected formula caused no change in the physical appearance of the formulation. Table 3(a) shows the *in-vitro* characterizations of the optimized formulation before and after sterilization and the *p* value obtained after statistical testing for the difference. All parameters measured showed a non-significant difference before and after sterilization (*p* < .05). The release profiles of the formulation before and after sterilization are shown in Figure 3(D). In order to determine the similarity between the release profiles of the optimized formulation before and after sterilization, similarity factor (*f*_2_) was calculated. A similarity exists when the calculated *f*_2_ value is between 50 and 100 (Flanner 1996), he calculated similarity factor is 62.59. This means that the two profiles are similar. These results prove that gamma sterilization is a suitable method for sterilization of the prepared formulation.

**Table 3.** (a) Results of the optimized formulation before and after sterilization.

	Entrapment efficiency (%)^a^	Particle size (nm)^a^	PDI^a^	Zeta Potential (mV)^a^	Q2h (%)^a^	Q8h (%)^a^	Q24h (%)^a^
Before sterilization	79.23 ± 1.12	810.95 ± 16.76	0.6785 ± 0.213	59.1 ± 0.99	40.984 ± 1.29	63.351 ± 1.61	91.113 ± 1.77
After sterilization	76.91 ± 0.486	1185 ± 210.72	0.602 ± 0.264	58.8 ± 0.42	43.911 ± 0.41	74.709 ± 5.28	92.42 ± 1.64
*p* Value	.076	.129	.780	.732	.050	.229	0.467

**Table:** 

(b) Comparison between optimized formulation and Alphagan ^®^P in graph parameters obtained after ocular administration.				
	%Dec. in IOP_max_^b^	T_max_^c^ (h)	AUC _0–24_^b^ (%.h)	MRT^b ^ (h)
Alphagan^®^ P	34.493 ± 3.601	2	81.454 ± 10.444	1.942 ± 0.251
Optimized formula	41.32 ± 2.42	2	409.215 ± 82.72	15.34 ± 4.20

**Table:** 

(c) Draize test.						
		Scores (*n* = 7)				
Group	Parameters	1 h	2 h	5 h	8 h	24 h
Optimized formula	Corneal	0	0	0	0	0
	Iris	0	0	0	0	0
	Conjunctiva	0	0	0	0	0
	Total score	0	0	0	0	0
						
Alphagan-P^®^	Corneal	0	0	0	0	0
	Iris	0	0	0	0	0
	Conjunctiva	1	0	0	0	0
	Total score	1	0	0	0	0

PDI: polydispersity index; Q2h, Q8h, Q24h: percentage of drug released after 2, 8, and 24 h respectively; % Dec. in IOP_max_: maximum percentage decrease in intraocular pressure; T_max_: time for maximum percentage decrease in intraocular pressure; AUC_0–24 h_: area under percentage decrease in intraocular pressure *vs.* time curve; MRT: mean residence time

^a^Data presented as mean ± SD (*n* = 3).

^b^Data presented as mean ± SD (*n* = 7).

^c^Data presented as median (*n* = 7).

#### Differential scanning calorimetry (DSC)

DSC is a conventional tool to investigate the physical nature of the material. DSC thermograms of pure BRT and the lyophilized optimized formulation are shown in Figure 2(II). The DSC thermogram of pure BRT demonstrates a sharp endothermic peak at 215.29 °C corresponding to its melting point (Aburahma & Mahmoud, 2011). This sharp peak indicates the crystallinity of BRT. Also, there is an exothermic peak at 262.55 °C indicating the thermal degradation of BRT (Morsi et al., 2014). The disappearance of BRT endothermic peaks in the thermogram of the lyophilized optimized formulation indicates complete dispersion of BRT in the amorphous state within the vesicles.

### *In-vivo* evaluation of the optimized formula

#### Ocular irritation evaluation

Draize test examined the ocular tolerability and safety of the topically applied niosomes versus Alphagan^®^P. As shown in Table 3(c), optimized formulation caused no irritation on the Draize scale of 0 to +3 during the whole study. The market BRT eye drops caused minor conjunctival irritation in the first hour in one rabbit. The irritation caused is mostly due to the preservatives present in the market eye drops, which led to reflex blinking and irritation (Singh et al., 2014). Lack of irritation caused by the tested formulation indicates the safety of ocular administration of it.

#### *In-vivo* pharmacodynamic study for the evaluation of IOP lowering effect of the optimized formula

The plot of the average percentage decrease in IOP (*n* = 7) as a function of time for Alphagan^®^P and the selected formula is shown in Figure 3(E). Evaluation parameters (% Dec. in IOP_max_, T_max_, AUC_0–24 h_, and MRT) were obtained from the graph and are represented in Table 3(b). Alphagan^®^P showed a maximum percentage decrease in IOP with a value of 34.49 ± 3.60% after 2 h then the IOP lowering effect is decreased gradually and returned to the baseline values after 5 h from ocular administration. While the selected formula showed a maximum percentage decrease in IOP with a value of 41.32 ± 2.42% after 2 h of ocular administration, the IOP lowering effect of the selected formula is sustained till the end of the study (for 24 h) with a value of 6.53 ± 2.92%. Both formulations showed the maximum decrease in IOP after 2 h (T_max_) then a gradual decrease in response was observed. Regarding the area under the percentage decrease in IOP response curve (AUC_0–24h_), it was statistically significantly higher for the tested formula when compared with Alphagan^®^P with a relative AUC_0–24h_ of 5.024. This means an improved ocular bioavailability of the tested formulation that could be due to the presence of the nonionic surfactant (Span 60), cholesterol, and lecithin which act as penetration enhancers and help in the diffusion of the drug to the cornea (Ammar et al., 2011; Khatoon et al., 2017). Also, the entrapment of BRT inside the vesicles guards against the degradation by the metabolic enzymes present in the tears and in the corneal epithelial surface thus, improving BRT ocular bioavailability (Dubey et al., 2014; Li et al., 2014). Regarding the MRT, there was a significant difference between the optimized formula and the market product with 7.90 folds increase in the MRT with the proniosomal formula. This means that the prepared formulation showed around eight times extension of the drug effect compared to the market product; this reveals the sustainment accomplished by the prepared proniosomal gel derived niosomes. This sustainment is assumed to be due to the increased ocular residence time of niosomes due to the mucoadhesive nature of it, and due to the sustainment of the drug release from the vesicles.

## Conclusions

Proniosomal gels of BRT were successfully prepared by coacervation phase separation method and could be considered as a promising ocular drug delivery vehicle for BRT in the treatment of glaucoma with a sustained release manner. Composition and *in-vitro* characterization of the prepared formulations in the preliminary screening and by the D-optimal design are represented in Table 1. Optimization of the formulation variables resulted in a formula with a desirability of 0.732. The results obtained after reconstitution of the optimized formula assured high entrapment of BRT and sustained release profile over 24 h. Gamma sterilization of the optimized formula causes no significant effect on the *in-vitro* characterizations, so it could be a suitable method for sterilization. TEM imaging of the optimized formula confirmed the typical spherical shape of the niosomes. Safety of ocular administration of the selected formula was assured by Draize test. The *in-vivo* pharmacodynamic study on New Zealand albino rabbits assured the accomplishment of the aim of our work. BRT-loaded proniosomal gels showed an improved ocular bioavailability and sustained drug release from the prepared vesicles.
